# Evidence of connections between cerebrospinal fluid and nasal lymphatic vessels in humans, non-human primates and other mammalian species

**DOI:** 10.1186/1743-8454-1-2

**Published:** 2004-12-10

**Authors:** Miles Johnston, Andrei Zakharov, Christina Papaiconomou, Giselle Salmasi, Dianna Armstrong

**Affiliations:** 1 Neuroscience Program, Department of Laboratory Medicine and Pathobiology, Sunnybrook and Women's College Health Sciences Centre, University of Toronto, 2075 Bayview Avenue, Toronto, Ontario, M4N 3M5, Canada

## Abstract

**Background:**

The parenchyma of the brain does not contain lymphatics. Consequently, it has been assumed that arachnoid projections into the cranial venous system are responsible for cerebrospinal fluid (CSF) absorption. However, recent quantitative and qualitative evidence in sheep suggest that nasal lymphatics have the major role in CSF transport. Nonetheless, the applicability of this concept to other species, especially to humans has never been clarified. The purpose of this study was to compare the CSF and nasal lymph associations in human and non-human primates with those observed in other mammalian species.

**Methods:**

Studies were performed in sheep, pigs, rabbits, rats, mice, monkeys and humans. Immediately after sacrifice (or up to 7 hours after death in humans), yellow Microfil was injected into the CSF compartment. The heads were cut in a sagittal plane.

**Results:**

In the seven species examined, Microfil was observed primarily in the subarachnoid space around the olfactory bulbs and cribriform plate. The contrast agent followed the olfactory nerves and entered extensive lymphatic networks in the submucosa associated with the olfactory and respiratory epithelium. This is the first direct evidence of the association between the CSF and nasal lymph compartments in humans.

**Conclusions:**

The fact that the pattern of Microfil distribution was similar in all species tested, suggested that CSF absorption into nasal lymphatics is a characteristic feature of all mammals including humans. It is tempting to speculate that some disorders of the CSF system (hydrocephalus and idiopathic intracranial hypertension for example) may relate either directly or indirectly to a lymphatic CSF absorption deficit.

## Background

The formation of CSF has been studied extensively [1] and the physical location at which it is formed within the ventricular system (the choroid plexus) has been well established. In contrast, while CSF absorption parameters have been investigated in many species, the mechanism and location of the transport sites is far from clear. It is assumed that projections of the arachnoid membrane into the cranial venous sinuses facilitate CSF removal however, there is very little convincing quantitative evidence to support a role for arachnoid villi and granulations in this process. Recent experiments suggest that the movement of CSF directly into the cranial venous system may only occur at high pressures suggesting that the arachnoid projections may have an accessory function rather than representing the primary locations at which CSF is absorbed [2,3].

The experimental evidence would seem to favour the view that a significant portion of CSF is removed from the subarachnoid space by nasal lymphatic vessels. This conclusion is based on experiments that span over 100 years of investigation [reviewed in [4]] with the most extensive analysis of CSF transport having been carried out more recently in sheep [2,3,5-17]. CSF convects through the cribriform plate into lymphatic vessels located in the submucosa associated with the olfactory and respiratory epithelium. The lymph within the ethmoidal lymphatics appears to be continuous with CSF in the perineurial spaces associated with the olfactory nerves. Multiple lymphatic ducts form a collar around the emerging nerve roots, which facilitates direct connection between CSF and lymph in the olfactory submucosa [18]. An extensive complex of lymphatic vessels in the ethmoid turbinal system conveys CSF through a variety of lymph nodes in the head and neck region to the cervical lymphatics that in turn, convey the CSF to the venous system [3].

There is also substantial data with various tracers and dyes that this lymphatic CSF transport pathway is a characteristic feature of many mammals [4]. However, there is still some uncertainty regarding the applicability of animal concepts to CSF transport in man. It has been argued that the larger brain, lack of sophisticated olfactory mechanisms and bipedal versus quadrapedal location make CSF transport in humans fundamentally different than that in animals.

There is no easy way to address the issue of lymphatic CSF transport in humans but recent experience visualizing CSF-lymph connections with Microfil, suggested that it may be possible to examine lymphatic CSF transport pathways in any species *post-mortem*. Microfil is a coloured, liquid silicone rubber compound which, when combined with a catalytic curing agent, polymerizes within 20 minutes. This agent facilitates the generation of 3 dimensional images of the spaces into which it has been injected. In a previous report, we infused Microfil into the subarachnoid compartment of sheep and noted that it was carried through the cribriform plate into an extensive network of lymphatic vessels in the nasal submucosa of this species [2,3,17,18]. Drawing on this experience, the main objective of the studies reported here was to examine potential CSF-nasal lymph pathways in a variety of species including human and non-human primates. The results of this investigation support the view that lymphatics play an important role in CSF transport in all mammalian species.

## Methods

### Animals

All animal experiments were approved by the ethics committee at Sunnybrook and Women's College Health Sciences Centre and conformed to the guidelines set by the Canadian Council on Animal Care and the Animals for Research Act of Ontario. Studies in the sheep, mouse, rat, rabbit and pig were performed at Sunnybrook and Women's College Health Sciences Centre. Studies on the Barbados green monkey were performed at the Barbados Primate Research Centre and Wildlife Reserve. Investigation of human specimens was approved by the Chief Coroner and the General Inspector of Anatomy for Ontario. These studies were conducted at the University of Toronto, Department of Surgery, Division of Anatomy.

### Experimental Objectives

The objective of these studies was to infuse a CSF tracer, Microfil (Flowtech, Mass), into the cranial subarachnoid space post-mortem to outline the pathways of CSF outflow. To accomplish this, the specimen was placed in sternal recumbancy with the head fixed in position. A laminectomy was performed on the upper regions of the spinal cord (generally between C1-C2) and the cisterna magna was cannulated with an angiocatheter or a silastic tube of appropriate size. A ligature was placed around the spinal cord and tightened to compress the spinal cord and meninges [13]. This ligature served two purposes. First, as we were interested in cranial absorption pathways, the amount of Microfil infused was less since this agent was prevented from passing into the spinal subarachnoid compartment during injection. Second, our experience suggested that infusion pressures effective in filling the pathways of interest were more easily achieved with the spinal CSF space negated.

With the exception of studies in the mice, all experiments were performed by infusing Microfil manually using a syringe. We did not routinely monitor infusion pressures post-mortem. However, in several of our initial studies in sheep, we measured infusion pressures between 200 and 300 cm H_2_O. Mice on the other hand appeared to be very fragile and injection by hand was problematic. In this species, we found that Microfil filling was more successful if this agent was introduced into the CSF compartment using a free-flow pressure system and reservoir as described below.

The best results were obtained using a Microfil preparation that was more dilute than that recommended in the product literature. To achieve this, 3 ml of diluent was used for every 1 ml of yellow Microfil^® ^(MV-122) and the material catalyzed with 10% (of total volume) of MV Curing Agent. In several sheep, blue Microfil (prepared as recommended in the instruction manual) was introduced into the vasculature at the same time that yellow Microfil was injected into the CSF compartment. The carotid arteries were catheterized and 20 ml of blue Microfil^® ^(MV-120) was infused into both arteries simultaneously. More specific details pertaining to each species is outlined below.

### Mice

Randomly bred, wild-type C57/Bl mice (*n *= 21, 16–19 gm; ~4–5 weeks old) were used for this investigation. They were fed lab rat chow (LabDiet 5001) until sacrifice. At the start of the preparation, the mice were euthanized. A laminectomy was performed at the C7 cervical-thoracic level of the vertebral column. Microfil was infused into the cranial subarachnoid space by one of two methods. In some preparations, Microfil was infused into the cranial subarachnoid space over a 5–10 minute interval through manual injection into an angiocatheter. In other experiments, the Microfil was administered using a free-flow pressure system with a reservoir filled with saline regulating the rate of flow into the cisterna magna. To achieve this, the three ports of a 3-way stopcock were attached to (a) a syringe containing Microfil, (b) a cannula attached to an angiocatheter inserted into the subarachnoid space, and (c) a cannula filled with saline hung vertically from a pole. The reservoir height was adjusted such that the saline solution exerted a pressure of approximately 100 cm H_2_0. Microfil was injected via the syringe until the agent reached the hub of the angiocatheter. At this point, the stopcock was adjusted to allow the column of PBS to exert a pressure on the Microfil, which pushed the contrast agent into the subarachnoid space.

### Rats

Randomly bred, Fischer 344 rats (*n *= 6, ~300 gm; 11–15 weeks old) were used for this investigation. They were fed lab rat chow (LabDiet 5001) until sacrifice. The rats were anaesthetized initially by placement in an induction chamber in which 5% halothane was administered for 2 minutes. Immediately after anesthesia, the rats were euthanized. A laminectomy was performed at the C7 cervical-thoracic level of the vertebral column. Microfil was infused into the cranial subarachnoid space over 5–10 minutes.

### Rabbits

Randomly bred, New Zealand white rabbits (*n *= 2, 3 and 4 kg; ~18 weeks old) were used for this investigation. They were fed rabbit chow (LabDiet 5321) until sacrifice. The rabbits were initially anesthetized with an intramuscular injection of Ketamine (50 mg/kg) and Rompun (5 mg/kg). Prior to surgery, the rabbits were euthanized. A laminectomy was performed at the C7 cervical-thoracic level of the vertebral column. Microfil was infused into the cranial subarachnoid space over a 5–10 minute period.

### Sheep

Randomly bred sheep (*n *= 29; 2.6–35 kg) including newborns (2–7 days old) and adult animals (6–8 months old) were used for this investigation. The neonatal lambs were bottle-fed formula (Lamb Replacement, Grober Inc. Cambridge, Ontario) until surgery. The lambs were anaesthetized initially by mask administration of incremental concentrations of halothane from 0.5 to 3%. The adult sheep were anesthetized initially by intravenous infusion of 2.5% sodium Pentothal solution. Subsequently, 1–3% halothane was delivered through an endotracheal tube via an A.D.S.1000 or Narkomed 2 respirator for surgical maintenance. In this species, a laminectomy was performed at the C1-C2 level under anesthesia followed by sacrifice. Microfil was infused into the cranial subarachnoid space over 10–15 minutes. In some animals, blue Microfil (prepared as recommended in the instruction manual) was introduced into the vasculature at the same time that yellow Microfil was injected into the CSF compartment. The carotid arteries were catheterized and 20 ml of blue Microfil^® ^(MV-120) was infused into both arteries simultaneously.

### Pigs

Randomly bred, Yorkshire pigs (*n *= 5, 20–25 kg; 8–9 weeks old) were used for this investigation. They were fed pellets (LabDiet Laboratory Animal Diet) until sacrifice. The pigs were anesthetized initially with an intramuscular injection of Ketamine (30 mg/kg) and Atropine (0.04 mg/kg). Surgical anesthesia was induced and maintained by 2–5% halothane delivered through an endotracheal tube via a Narkomed 2 respirator. Prior to surgery, the pigs were euthanized. A laminectomy was performed at the C1-C2 level and Microfil was infused into the cranial subarachnoid space over a 10–15 minute period.

### Monkeys

Randomly bred, Barbados green monkeys (*Cercopithecus aethiops sabeus*) (*n *= 6, 4.0–5.6 kg; ~6 years old) were used for this investigation. They were fed twice daily various natural foods until sacrifice. At the start of the preparation, the monkeys were anaesthetized with Xylazine/Ketamine (100 mg/kg) injection. Subsequently, the monkeys were euthanized. A laminectomy between C7 and T1 was performed and Microfil was infused into the cranial subarachnoid space over a 5–10 minute period.

### Humans

Three recently deceased cadaveric preparations were examined (3 females, 77, 80 and 90 yrs old). Access to the cadaveric material was permitted between 6 and 7 hours post mortem. A laminectomy between C7 and T1 was performed and Microfil was infused into the cranial subarachnoid space.

### Analysis of tissues

Following injection with Microfil, the heads of mice, rats, rabbits, sheep and pigs were stored at 4°C overnight. The next day, the heads were skinned, sectioned in a sagittal orientation and fixed in 10% formalin. In the monkey studies, the Microfil was allowed to set for 2 hours after injection followed by sectioning and fixation with formalin (2–3 weeks) and 70% alcohol (immediately before shipment to Canada). After Microfil infusion in humans, the heads were placed in a freezer for several days (-18°C) after which they were sectioned and fixed with Kaiserling's solution for at least 5 days before dissection.

Sectioning of the fixed tissues was made in several orientations including coronal cuts anterior and posterior to the cribriform plate and/or sagittal sections. The various tissues were dissected as appropriate under a dissecting microscope (Leica M651, Wild Leizt; EMZ-TR, Meiji Techno; Fisher Stereomaster) and images were captured on a Nikon digital camera (Coolpix 995). All images presented in this report are in the midsagittal plane from the cut surfaces of the heads.

## Results

### Technical Issues

Filling of the subarachnoid compartment and extension of the Microfil into the lymphatic vessels of the olfactory and respiratory submucosa was dependent on several factors including the size of the species and the time of injection after death. Filling of the CSF space and attendant lymphatic vessels with Microfil was easiest in the larger animals and was increasingly challenging as the species declined in size. Additionally, we were able to achieve successful filling up to 6 hours after death but the best results were obtained when the agent was injected immediately after the animal was euthanized. For reasons unknown, in a few unsuccessful preparations the Microfil did not cure correctly.

The number of human specimens was limited to 3. In one attempt, there was incomplete filling of the subarachnoid space due to rupture of the meninges and dura. In another preparation, a large metastatic tumor located in the frontal lobe with accompanying perifocal edema and petechial hemorrhages caused significant dislocation of the brain and prevented filling of the CSF spaces. In a third preparation, where the cause of death was not connected to brain disease (lung cancer), the infusion was successful. Table 1 summarizes the experimental details and the success rates of the Microfil infusions in the various species.

**Table 1:** Details of animal studies

Species	Number of animals used	Number of successful preparations	Hours after death	Injected Microfil volume (ml)
mice	21	7	0–1	1–1.5
rats	6	4	0–1	2.5–3
rabbits	2	2	0–1	10
sheep	29	26	0–1	20–25
pigs	5	2*	2–72	20–25
monkeys	6	5	0–1	20
humans	3	1**	5–7	60–180

* Successful filling was achieved 2 and 5 hours after death

** In the one successful human preparation, 180 ml of Microfil was infused

### CSF Transport Pathways

Microfil was observed throughout the subarachnoid compartment associated with the base of the brain and generally filled the basal cisterns in all species. Microfil was also observed commonly in the subarachnoid space over the convexities of the brain.

**Figure 1:**
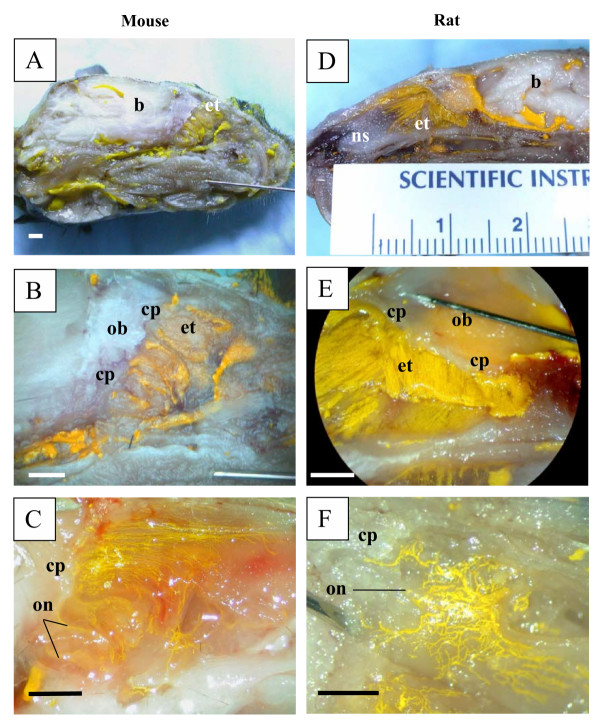
Microfil distribution patterns in the head of mice and rats. All images are presented in sagittal plane with gradual magnification of the olfactory area adjacent to the cribriform plate. Reference scales are provided either as a ruler in the image (mm) or as a longitudinal bar (1 mm). A-C illustrates images of the mouse and D-F images of rat. The Microfil can be viewed in the perineurial space of the olfactory nerves external to the cranium and a network of lymphatic vessels containing yellow Microfil can be observed in the ethmoid turbinal systems of both species. In the example illustrated in 1C, the image was captured before fixation. The reddish spots are areas of hemorrhage in this particular animal. b – brain; cp – cribriform plate; et – ethmoid turbinates; ob – olfactory bulbs; on – olfactory nerves; ns – nasal septum.

**Figure 2:**
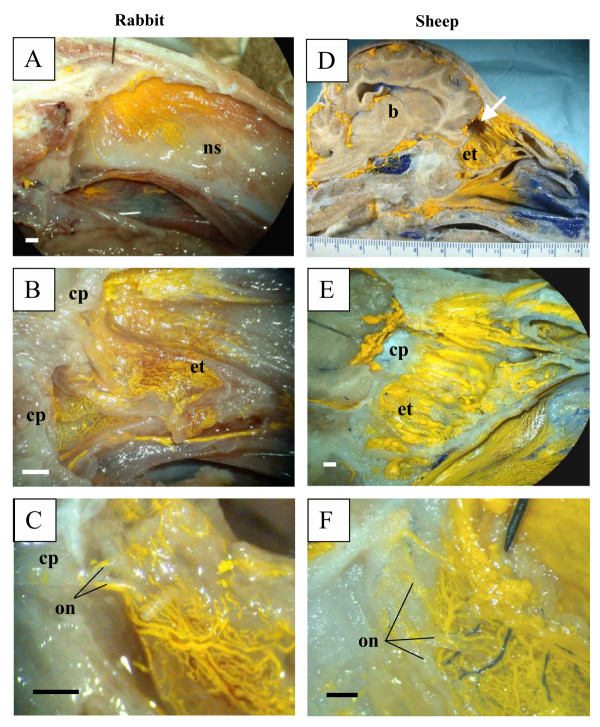
Microfil distribution patterns in the head of rabbits and sheep. All images are presented in sagittal plane with gradual magnification of the olfactory area adjacent to the cribriform plate. Reference scales are provided either as a ruler in the image (mm) or as a longitudinal bar (1 mm). A-C illustrates images of the rabbit and D-F images of sheep. In both species, the Microfil can be viewed in the perineurial spaces of the olfactory nerves external to the cranium, which merge into network of lymphatic vessels in the ethmoid turbinal systems. b – brain; cp – cribriform plate; et – ethmoid turbinates; on – olfactory nerves; ns – nasal septum; arrow in D – portion of cribriform plate removed for histology.

**Figure 3:**
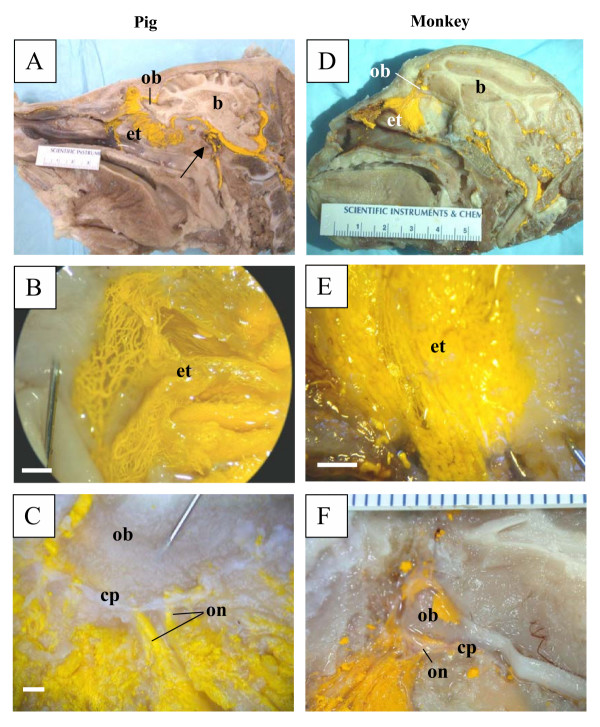
Microfil distribution patterns in the head of pigs and Barbados green monkeys. All images are presented in sagittal plane with gradual magnification of the olfactory area adjacent to the cribriform plate. Reference scales are provided either as a ruler in the image (mm) or as a longitudinal bar (1 mm). A-C illustrates images of the pig and D-F images of the monkey. In both species, the Microfil can be viewed in the perineurial spaces of the olfactory nerves external to the cranium, which merge into network of lymphatic vessels in the ethmoid turbinal systems. In A, Microfil can be observed penetrating the dura and entering the cavernous sinus with partial filling of the retromandibular vein (arrow). b – brain; cp – cribriform plate; et – ethmoid turbinates; ob – olfactory bulbs; on – olfactory nerves.

**Figure 4:**
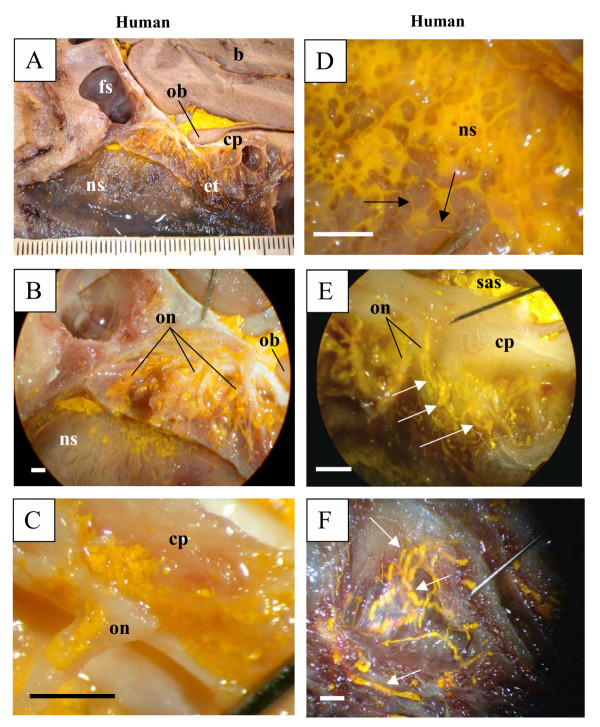
Microfil distribution patterns in the head of a human (A-F). All images are presented in sagittal plane with gradual magnification of the olfactory area adjacent to the cribriform plate. Reference scales are provided either as a ruler in the image (mm) or as a longitudinal bar (1 mm). As in the other species, Microfil introduced into the subarachnoid space was observed around the olfactory bulb (A), in the perineurial spaces of the olfactory nerves (B, C) and in the lymphatics of the nasal septum (D), ethmoid labyrinth (E) and superior turbinate (F). Due to tissue deterioration, some of the lymphatic vessels had ruptured and Microfil was noted in the interstitium of the submucosa of the nasal septum (D). In (E), Microfil is observed in the subarachnoid space and the perineurial space of olfactory nerves. The perineurial Microfil is continuous with that in lymphatic vessels (arrows). Intact lymphatic vessels containing Microfil are outlined with arrows (D, E, F). b – brain; fs – frontal sinus; cp – cribriform plate; et – ethmoid turbinates; ob – olfactory bulbs; on – olfactory nerves; ns – nasal septum; sas – subarachnoid space.

**Figure 5:**
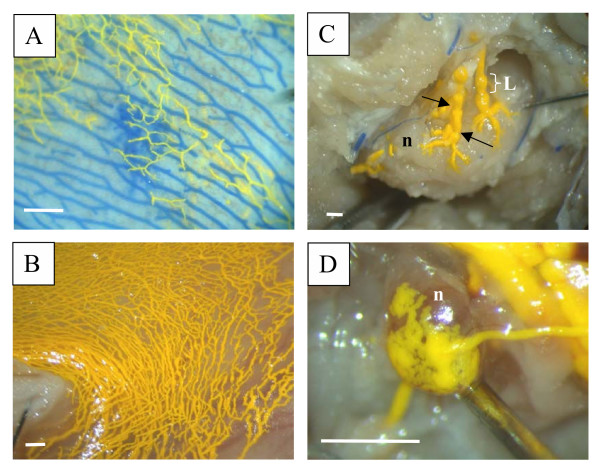
Visualization of lymphatic vessels containing Microfil. Reference scales are provided as a longitudinal bar (1 mm). A – illustrates the ability to separate blood vessels (blue) from lymphatics (yellow) using differently coloured Microfil preparations (sheep). B – demonstrates lymphatic networks that have taken up Microfil that was injected into the subarachnoid compartment (pig). These networks ultimately connect with various lymph nodes (C – retropharyngeal node example in sheep; D – submandibular node example in mouse). The prenodal vessels can be visualized converging on the lymph nodes with the lymphatics congregating into a labyrinth of small ducts or foot processes on the node capsule. Blood vessels containing blue Microfil can be observed proximal to the node in C. The functional contractile unit of lymphatic vessels is the lymphangion, which is a segment of vessel between 2 valves (valves illustrated by arrows in C). n – lymph node; L – lymphangion.

In all species examined in this report (mouse and rat, Figure 1; rabbit and sheep, Figure 2; pig and monkey, Figure 3; and human, Figure 4), Microfil could be observed in a patchy distribution along the olfactory nerves external to the cranium and in lymphatics associated with the submucosa of the olfactory epithelium. More extensive lymphatic networks containing Microfil were observed in the submucosa of the ethmoid labyrinth and adjacent nasal septum. In some of the sheep preparations, blue Microfil was introduced into the vascular system and yellow Microfil into the subarachnoid space. Yellow lymphatic vessels were easily distinguished from blue arterioles (Figure 5A).

The structural properties of the ethmoid labyrinth determined the density of distribution of Microfil into lymphatics of the nasal cavity. The rat, mouse, rabbit, sheep, and pig have large olfactory bulbs and a broad, deep olfactory fosse in the cribriform plate. The structure of the ethmoid bone determines the broad distribution of olfactory nerves from the midline to the latero-posterior nose. In monkeys and humans, the ethmoid bone is more delicate and the olfactory nerves are concentrated along the midline. The exits of olfactory nerves from the skull base were filled with Microfil and in most successful preparations it was possible to see the trunks of the olfactory nerves with Microfil distributed in the perineurial space.

We reported previously that Microfil was observed primarily within lymphatic vessels in the sheep with very little evidence of this agent within the interstitium of the submucosa [17,18]. While we did not investigate the tissues with histological techniques in this report, this also seemed to be the case in all of the species examined except for the human specimens. This was most likely due to the fact that we were able to inject the Microfil immediately or shortly after death in the animals. In humans however, there was a delay in getting access to the cadavers with the result that there was some degree of deterioration of the tissues. From previous studies in which Microfil was injected into animals at various times after sacrifice, we know that the CSF transport pathways into lymphatics degenerate rapidly after death and the filling of the vessels with Microfil is compromised. In any event, while many vessels had ruptured in the human tissues, Microfil was observed in enough intact lymphatic vessels to confirm the principle of CSF-nasal lymph connections in man.

It has been well documented that CSF transports through the cribriform plate into nasal lymphatics which progress downstream to various lymph nodes including retropharyngeal, cervical, submandibular and preauricular lymph nodes. In some preparations in sheep and mice we were able to follow Microfil-containing lymphatic networks to the point at which they converged on these lymph nodes (examples are illustrated in Figure 5C,5D).

Some Microfil was observed in various veins in the head and neck region external to the cranium. An example can be seen in Figure 3A in the pig. In this case the retromandibular vein was partially filled with Microfil. Additionally, in some cases, Microfil was observed to enter the venous sinuses within the cranium.

In some of the preparations in all species, Microfil was observed lying freely in the nasal cavity. This material lay proximal to terminal branches of the olfactory nerves and supports the concept of physiological and post inflammatory CSF rhinorrhea.

## Discussion

Nasal lymphatic vessels have been implicated in CSF transport for many years. However, the concept has not received mainstream acceptance for several reasons.

First, the data supporting lymphatic function has been based entirely on animal studies especially the sheep. However, histological and radiological evidence indicate that CSF-lymph connections exist in other species including rats [8,19-24], mice [25], rabbits [26-28], cats [20,29,30], dogs [20,30], guinea pigs [31], and non-human primates [30,32]. In the latter case, injection of radioactive albumin into the CSF compartment of monkeys led to elevated concentrations of tracer in the cervical lymph nodes [32].

Circumstantial evidence also suggests that similar CSF-lymphatic connections exist in humans as well [22,33-36]. For example, Indian ink administered into the CSF in human autopsy material [35] was observed to fill the perineurial spaces around the olfactory nerve branches and was found in the nasal submucosal tissue. Similarly, in an individual with subarachnoid hemorrhage, red blood cells were observed around the olfactory nerves and within the nasal mucosa. However, until now, direct evidence that the CSF and lymph compartments were linked in human and non-human primates was lacking.

The Microfil images presented in this report demonstrate that the passage of CSF along the olfactory nerves into lymphatics is a characteristic feature of several mammalian species. The Microfil distribution patterns from the mouse to the human were remarkably similar. It must be acknowledged that we had limited access to human material and that studies with the cadaveric material are ongoing. However, the fact that Microfil was observed external to the cranium in the nasal submucosa adjacent to the cribriform plate in both human and Barbados green monkey specimens supports the view that CSF absorption in primates is not significantly different (at least qualitatively) from that in other mammalian species.

In a previous study, we injected yellow Microfil into the subarachnoid space and blue Microfil into the carotid arteries [18]. This protocol permitted the separation of the blood vessels from lymphatics without the use of promiscuous molecular markers. Indeed, the larger Microfil-filled lymphatic vessels demonstrated the classical 'lymphangion' structure with constrictions of the vessels where the valves were positioned (example in Fig 5C). The vessels could be traced in some instances to prenodal ducts that converged on various lymph nodes [18]. In the study reported here, we have included examples of this in sheep and mice (Figure 5C,5D).

Studies with Microfil provide valuable information on 'potential' CSF transport routes but one must be cautious in applying post-mortem observations to physiological processes. However, in sheep there is considerable quantitative data that supports the concept that the cribriform-olfactory nerve-lymphatic pathway contributes significantly to volumetric CSF absorption [2,3,5-9,11,13-16]. Given that the Microfil distribution patterns in sheep, pigs, rabbits, rats, mice, Barbados green monkeys and humans appear remarkably similar, it would seem likely that this pathway is important for CSF clearance in each of these species.

Another reason for the skepticism regarding the role of lymphatics in CSF transport is the generally held view that arachnoid projections function in this role. However, the quantitative evidence supporting a role for arachnoid projections is very limited and unconvincing [37-39]. It must be acknowledged that we observed Microfil within a variety of veins external to the cranium and in the dural sinuses. With regards to the former, Microfil entry was due (at least in part) to passage of the contrast agent through the dura at the base of the brain into the cavernous sinus. The Microfil appeared to push into this venous system and fill several veins. Whether this observation has any physiological relevance is unknown.

The route by which Microfil gains entry into the dural venous sinuses is unknown but is currently under investigation. However, the concept of direct CSF transport into the superior sagittal sinus continues to be problematic. We used several experimental approaches to assess this issue in previous studies in sheep. The most informative technique was to employ the classical approach of Mann to look for enrichment of a radioactive CSF tracer in the superior sagittal sinus compared to a peripheral vein [40]. The Mann group observed that the concentration of soluble and particulate tracer was considerably greater in the sinus blood than in the peripheral circulation (inulin concentrations were up to 30 times greater). However, the peak CSF pressures achieved during bolus infusions were greater than 80 cm H_2_O, a level of pressure that is clearly pathological. Other groups have failed to observe enrichment of a CSF tracer in the superior sagittal sinus of the rabbit, cat and monkey when the intracranial pressures achieved in the experiments simulated physiological conditions more realistically [32,41,42].

In our investigations in adult sheep, at a CSF-superior sagittal sinus pressure gradient of around 10 cm H_2_O (normal gradient ~5 cm H_2_O) no enrichment was observed. When the gradient favouring absorption was greater than 20 cm H_2_O, a modest enrichment of tracer was noted [3].

A similar result was obtained with tracer enrichment experiments in newborn lambs. The latter result was surprising. Adult sheep have abundant arachnoid projections in the superior sagittal sinus which are visible when stained with Evans blue dye but, in the neonatal lamb, arachnoid projections are few or absent [2]. Therefore, tracer transport into the superior sagittal sinus could not be correlated with the presence or absence of arachnoid projections although some transport directly into the cranial venous system seemed to occur.

In our studies the maximum enrichment of CSF tracer in the superior sagittal sinus was observed only within the first few minutes after increasing intracranial pressure [3]. Within a short time, the tracer concentrations in the sagittal sinus and peripheral venous blood were equal. This suggested the possibility that the tracer enrichment method outlined above has the potential to underestimate the role of arachnoid projections since vigorous tracer transport to peripheral veins by some other mechanism could cancel out any perceived tracer enrichment in the cranial venous sinuses. Projections of the arachnoid membrane into the veins at the base of the brain have been described but at this time, there is no evidence that these structures play a role in CSF transport. The most obvious contributor to the peripheral venous tracer would be the lymphatic circulation.

To determine if the lack of CSF tracer enrichment in the superior sagittal sinus was due to lymphatic transport into peripheral veins, we created what we thought would be the conditions most favourable to measure clearance via the arachnoid granulations. We sealed the cribriform plate and prevented CSF transport into the spinal subarachnoid compartment thus negating much of the lymphatic transport capacity. Nonetheless, even under these conditions when the CSF-superior sagittal sinus pressure gradient was raised to 20 cm H_2_O (~4 fold higher than normal), no enrichment was observed. At pressure gradients greater than this level, the concentration of the tracer was significantly higher than in the peripheral circulation. Therefore, the lack of enrichment of the CSF tracer in the superior sagittal sinus at normal physiological intracranial pressures does not appear to be due to downstream lymphatic transport of the CSF tracer. Contrary to the 'classical' view, direct transport of CSF into cranial veins (whether this occurs through arachnoid projections we cannot determine unequivocally) may represent an auxiliary system that is recruited when intracranial pressures are high or when other transport pathways are compromised.

In summary, we have never been able to obtain persuasive evidence that direct CSF transport into the venous system is a physiological reality. In contrast, the qualitative and quantitative support for nasal lymphatic role in CSF absorption is gaining momentum.

## Conclusions

In this report, we present the first direct evidence for CSF-lymph connections in primates including monkeys and one human. The fact that the Microfil distribution patterns were very similar in the seven species tested suggested that the lymphatic transport of CSF represented an important pathway by which CSF is cleared from the subarachnoid space in mammalian species. A workable hypothesis can be developed around the concept that defects in lymphatic CSF transport may contribute to syndromes characterized by elevated intracranial pressure and/or hydrocephalus.

## Competing Interests

The author(s) declare that they have no competing interests.

## Author's Contributions

MJ: conceived of the study, and participated in its design and coordination.

AZ: developed the application of the Microfil method to the analysis of CSF transport pathways, participated in all experiments, created an image catalogue and helped in the preparation of the manuscript.

CP: aided in the experiments in human, sheep, rats and mice and helped in the preparation of the manuscript

GS: performed the studies in rats and mice

DA: in conjunction with AZ performed the studies in humans and monkeys and assisted in all experiments.

All authors read and approved the final manuscript.
